# Reconstitution of Functional Integrin αIIbβ3 and Its Activation in Plasma Membrane-Mimetic Lipid Environments

**DOI:** 10.3390/membranes11070499

**Published:** 2021-06-30

**Authors:** Una Janke, Alexandra Mitlehner, Aileen Weide, Theresia Gutmann, Mihaela Delcea

**Affiliations:** 1 Institute of Biochemistry, University of Greifswald, Felix-Hausdorff-Straße 4, 17489 Greifswald, Germany; una.janke@uni-greifswald.de (U.J.); alexandramagdalena.mitlehner@stud.uni-greifswald.de (A.M.); aileen.weide@stud.uni-greifswald.de (A.W.); 2 ZIK HIKE-Zentrum für Innovationskompetenz “Humorale Immunreaktionen bei Kardiovaskulären Erkrankungen”, University of Greifswald, Fleischmannstraße 42, 17489 Greifswald, Germany; 3 DZHK (German Centre for Cardiovascular Research), Partner Site Greifswald, 17489 Greifswald, Germany; 4 Paul Langerhans Institute Dresden of the Helmholtz Zentrum München at the University Hospital and Faculty of Medicine Carl Gustav Carus of TU Dresden, 01307 Dresden, Germany; theresia.gutmann@tu-dresden.de

**Keywords:** liposomes, lipids, integrin αIIbβ3, reconstitution, detergents

## Abstract

The study of the platelet receptor integrin αIIbβ3 in a membrane-mimetic environment without interfering signalling pathways is crucial to understand protein structure and dynamics. Our understanding of this receptor and its sequential activation steps has been tremendously progressing using structural and reconstitution approaches in model membranes, such as liposomes or supported-lipid bilayers. For most αIIbβ3 reconstitution approaches, saturated short-chain lipids have been used, which is not reflecting the native platelet cell membrane composition. We report here on the reconstitution of label-free full-length αIIbβ3 in liposomes containing cholesterol, sphingomyelin, and unsaturated phosphatidylcholine mimicking the plasma membrane that formed supported-lipid bilayers for quartz-crystal microbalance with dissipation (QCM-D) experiments. We demonstrate the relevance of the lipid environment and its resulting physicochemical properties on integrin reconstitution efficiency and its conformational dynamics. We present here an approach to investigate αIIbβ3 in a biomimetic membrane system as a useful platform do dissect disease-relevant integrin mutations and effects on ligand binding in a lipid-specific context, which might be applicable for drug screening.

## 1. Introduction

Platelets are specialized blood cells with central functions in hemostasis, blood coagulation, wound healing, inflammation, and cancer development [1]. Their immediate responsiveness to extracellular cues is mediated by adhesion and signaling molecules, with the heterodimeric integrin αIIbβ3 being the most abundant platelet receptor [2]. Upon binding to extracellular cell matrix components, integrin receptors recruit heterogeneous multiprotein complexes to their cytoplasmic tails tethering them to the actin cytoskeleton. Integrins thus, constitute a direct bridge between the extracellular matrix and the cytoskeleton providing mechanosensation and adhesion [3]. Integrin αIIbβ3 function has been directly related to distinct conformations (i.e., closed/bent, intermediate-extended, and ligand-occupied state) that dynamically regulate the platelet activation and ligand binding resulting in platelet aggregation [4,5,6]. 

To understand its structural characteristics and conformational changes, it is essential to study the receptor in isolation without interfering associated proteins or signalling pathways involving protein and lipid turnover [7]. Reconstitution of this membrane-spanning receptor in model membranes, such as liposomes or supported lipid bilayers (SLB), enables the investigation under well-controlled conditions, which was the subject of several publications in the last years [5,8,9,10,11,12,13,14]. These studies focused mainly on the integrin structure and its dynamic activation process, while few examples investigated the potential impact of the lipid bilayer and its composition, e.g., the stabilization of αIIbβ3 transmembrane domains by anionic lipids [15] or fibrinogen-induced integrin clustering in specific membrane domains [16]. 

The generation of an appropriate membrane-mimetic in vitro reconstitution system ensuring efficient transmembrane protein insertion and retaining its functionality is not trivial [17]. Furthermost, in vitro transmembrane protein reconstitution approaches involve the extraction and purification of the protein-of-interest using detergents, which is then added to detergent-destabilized liposomes followed by detergent removal and separation from non-embedded protein [18,19]. The most crucial parameters to consider are the lipid composition including emerging membrane properties, the lipid to protein ratio, the choice and concentration of detergents, and the method of detergent removal. Traditionally, the non-ionic detergent Triton X-100 has been used to purify integrins, owing to its high solubilisation efficiency while retaining integrin functionality in terms of ligand binding [5,9,11,20]. Its low critical micellar concentration and large micelle size prevents Triton X-100 to be efficiently removed from detergent-lipid-protein solutions by dialysis [19], hence SM2 biobead adsorption has been widely used to generate proteoliposomes. Most αIIbβ3 integrin reconstitution approaches involved exclusively lipids with short saturated acyl chains, in particular, 1,2-dimyristoyl-sn-glycero-3-phosphocholine (DMPC) and 1,2-dimyristoyl-sn-glycero-3-phospho-rac-1-glycerol (DMPG) [9,11,12,20,21].

We have previously shown that αIIbβ3 integrins embedded in DMPG:DMPC proteoliposomes are amenable to activation studies by quartz crystal microbalance with dissipation monitoring (QCM-D) [10,22]. This sensitive technology enables us to study membrane-embedded proteins in an entirely label-free manner providing real-time information about mass adsorption, conformational changes, and viscoelastic properties [23]. For QCM-D studies, membrane proteins are first embedded in lipid vesicles that are used to form SLBs on the crystal surface.

However, the previously used DMPG:DMPC lipid composition and the resulting membrane properties are in stark contrast to lipid signatures of animal cell membranes [24,25]. In particular, the integrin transmembrane domains, which are transmitting the bidirectional integrin activation processes, are directly affected by the membrane properties, such as thickness, lateral pressure, and fluidity [17,26,27]. 

Here, we report the adaption of αIIbβ3 reconstitution protocol for QCM-D measurements in lipid environments that more closely resemble the plasma membrane. To mimic the external leaflet of the platelet plasma membrane, we used a ternary lipid system comprising cholesterol, sphingomyelin (SM) and an unsaturated phospholipid represented by 1,2-dioleoyl-sn-glycero-3-phosphocholine (DOPC) [25,28]. Modulating their respective ratios, we are able to tune the membrane properties based on available phase diagrams [29,30,31] to more fluid liquid disordered (Ld) phases, rigid liquid-ordered (Lo) phases or a coexistence of both (Ld/Lo) [9]. 

Based on our previous study [10], we first switched the lipid composition to DOPC, SM, and cholesterol using Triton X-100-solubilised protein and detergent removal by SM2 biobeads, which we call the “biobead protocol” throughout. Proteoliposomes generated by this procedure were highly heterogeneous, prone to clumping, and–most disappointingly–never formed homogeneous lipid bilayers on the quartz crystal preventing QCM-D measurements.

Therefore, the αIIbβ3 reconstitution protocol was further modified, using a detergent exchange to CHAPS and slow detergent removal by dialysis [19]. This zwitterionic detergent has protein disaggregating, non-disordering properties, a steroid-type chemical structure [32,33] as well as a high critical micellar concentration facilitating its removal by dialysis [34]. This adapted protocol was applicable to several lipid compositions of distinct physicochemical properties, which we demonstrated here using different amounts of cholesterol. Remarkably, integrin αIIbβ3 could be efficiently activated as observed by the Mn^2+^-induced conformational change towards the extended conformation, which appeared to be sensitive to the cholesterol content and lipid order of its environment. 

## 2. Materials and Methods

**Chemicals, Lipids and Proteins**

Human integrin αIIbβ3 was purchased from Enzyme Research Laboratories (South Bend, IN, USA). 1,2-dioleoyl-sn-glycero-3-phosphocholine (DOPC), N-stearoyl-D-erythro-sphingosylphosphorylcholine (SM), and cholesterol (ovine) were obtained from Avanti Polar Lipids Inc. (Alabaster, AL, USA). SM2 biobeads were supplied by Bio-Rad (Munich, Germany). Tris-Base and NaCl were bought from Sigma-Aldrich (Taufkirchen, Germany). CaCl_2_, MnCl_2_, CHAPS, 2′,7′-dichlorofluorescein, methyl α-D mannopyranoside, Triton X-100, 4-(2-hydroxyethyl)-1-piperazineethanesulfonic acid (HEPES), and methanol were purchased from Carl Roth GmbH (Karlsruhe, Germany). Sucrose and sodium dodecyl sulfate (SDS) were obtained from Merck KgaA (Darmstadt, Germany). Phenylmethylsulfonyl fluoride (PMSF) and MgCl_2_ were purchased from AppliChem GmbH (Darmstadt, Germany). All columns were supplied by Cytiva formerly GE Healthcare (Freiburg, Germany).

**Integrin Purification**

***Platelet Lysate Preparation.*** Outdated and concentrated human platelets (500 mL) were obtained from the AG Cardiovascular Cell Research at the University Medicine Greifswald and experiments were performed in accordance with the ethical guidelines of the Declaration of Helsinki. Platelets were pelleted at 1800× *g* and room temperature (RT) for 30 min. The cell pellet was washed with 20 mM Tris, pH 7.4, 150 mM NaCl and then dissolved in 50 mL lysis buffer (20 mM Tris, pH 7.4, 150 mM NaCl, 0.5 mM CaCl_2_, 5 mM PMSF, 10 µmol Leupeptin [Thermo Fischer Scientific, Waltham, MA, USA], 1% Triton X-100) and stirred overnight at 4 °C. The lysate was then filtered through a 0.45 µM mixed cellulose ester membrane filter (Merck KgA, Darmstadt, Germany). All subsequent purification steps have been carried out at RT. Representative chromatograms and SDS-PAGE gels are presented in Figure S1.

***Concanavalin A (ConA) Column***. After equilibration of the HiTrap ConA 4B 5mL column at a flow rate of 1 mL/min with running buffer (20 mM Tris, pH7.4, 150 mM NaCl, 1 mM CaCl_2_, 1 mM MgCl_2_, 0.1% Triton X-100), the filtered cell lysate was loaded onto the column with a flow rate of 1 mL/min. To exchange the Triton X-100 detergent to CHAPS, the column was washed with at least ten column volumes of 20 mM HEPES, pH 7.4, 150 mM NaCl, 1 mM CaCl_2_, 1 mM MgCl_2_, 1% CHAPS. An integrin-enriched fraction eluted with 20 mM HEPES, pH 7.4, 150 mM NaCl, 1 mM CaCl_2_, 1 mM MgCl_2_, 1% CHAPS, 5 μmol Leupeptin, 200 mM methyl α-D mannopyranoside. Elution fractions containing integrin detected by SDS-PAGE and Coomassie staining were pooled.

***Heparin Column.*** To eliminate fibrinogen and thrombospondin-1 from the sample, the pooled ConA eluate was passed through a HiTrap Heparin HP 5 mL column using a flow rate of 1 mL/min after equilibration with 20 mM HEPES, pH 7.4, 150 mM NaCl, 1 mM CaCl_2_, 1 mM MgCl_2_, 1% CHAPS. The flow through was then concentrated to a concentration of approximately 2 mg/mL in a 30 KDa cut-off Amicon Ultra-15 Centrifugal Filter Unit (Merck KgaA, Darmstadt, Germany). 

***Size Exclusion Chromatography***. αIIbβ3 was further purified by size exclusion chromatography with a HiPrep 16/60 Sephacryl S-300 column equilibrated in 20 mM HEPES, pH 7.4, 150 mM NaCl, 1 mM CaCl_2_, 1 mM MgCl_2_, 1% CHAPS operated at 0.5 mL/min. The peak fractions were analysed by SDS-PAGE, integrin-containing fractions were pooled, and concentrated to approximately 2 mg/mL with a 30 KDa cut-off Amicon Ultra-15 Centrifugal Filter Unit. The protein amount was determined by bicinchonic acid assay (BCA) kit (Sigma Aldrich, Taufkirchen, Germany) following the manufacturers instruction and samples were stored at −80 °C. Thermostability was confirmed by nano-differential scanning fluorimetry (nanoDSF) using a Prometheus NT.48 instrument with backscattering optics (NanoTemper Technologies, Munich, Germany). αIIbβ3 was diluted to 1.26 mg/mL with 25 mM HEPES, pH 7.4, 150 mM NaCl, 1 mM CaCl_2_, 1% CHAPS, loaded into standard capillaries (NanoTemper Technologies), and unfolding was monitored recording the intrinsic fluorescence at 350 and 330 nm during a temperature ramp (20–90 °C, °C/min) at 30% LED power.

**Reconstitution of Integrin αIIbβ3**

Integrin αIIbβ3 was embedded in lipid vesicles containing various ratios of the lipids DOPC, SM, and cholesterol using two fundamentally different reconstitution approaches, which we refer to here as “biobead protocol” and “dialysis protocol”.

***Biobead Protocol.*** The preparation of proteoliposomes followed an adapted protocol from Janke et al. [10], on the basis of the work from Erb and Engel [8]. A total amount of 1.6 µmol DOPC, SM, and cholesterol in a molar ratio of 45:30:25 (referred to as Ld/Lo composition) was dried under a stream of nitrogen and left in vacuum overnight. Afterwards, lipids were dissolved in 1 mL biobead buffer (20 mM Tris, pH 7.4, 50 mM NaCl, and 1 mM CaCl_2_) containing 0.1% Triton X-100 and 0.5 mg of αIIbβ3 (Enzyme Research Laboratories) buffer and incubated for 2 h at 37 °C. The initial protein:lipid ratio amounts was 1:800. Triton X-100 was removed by adding 70 mg SM-2 biobeads for 3 h at 37 °C twice. To isolate non-reconstituted protein from the proteoliposomes, the sample was loaded on a 5 mL four-step sucrose gradient (2 M, 1.2 M, 0.8 M, 0.4 M sucrose in biobead buffer) and ultracentrifuged at 4 °C and 268,000× *g* for 24 h (MLS-50 swinging-bucket rotor, Beckmann Coulter, Brea, CA, USA). The fraction containing proteoliposomes was dialyzed for three days against biobead buffer. Empty liposomes treated the same way (adding protein storage buffer instead of purified protein) served as controls. The proteoliposomes and empty liposomes were stored at 4 °C and used for experiments within four days. The reconstitution efficiency (i.e., a proxy of the amount of protein embedded in liposomes as assessed by SDS-PAGE with the final sample versus initially added protein) was approximately 25%.

***Dialysis Protocol.*** The reconstitution of integrin αIIbβ3 into liposomes followed a protocol adapted from Coskun et al. [35]. 1.6 µmol lipids consisting of DOPC, SM, and cholesterol (45:30:25 mol% or as indicated in the respective figures), was dried under a stream of nitrogen and left in vacuum overnight. Lipid films were hydrated with 1 mL dialysis buffer (25 mM HEPES, pH 7.4, 150 mM NaCl, 1 mM CaCl_2_) to obtain a total lipid concentration of 1.6 µM and incubated vigorously shaking for 20 min at 54 °C. Unilamellar vesicles were formed by ten freeze-thaw cycles (liquid nitrogen–54 °C) and sized by extrusion with an extruder (Avanti Polar Lipids Inc, Alabaster, AL, USA) at 54 °C through a 100 nm pore size membrane. The liposomes were destabilized by adding 1.6% CHAPS and their size was monitored once by DLS and turbidity measurements. 0.5 mg purified αIIbβ3 was added and incubated for 20 min at 30 °C at 450 rpm in a Thermomixer (Eppendorf AG, Hamburg, Germany). The sample was then dialyzed four times against 2 L of dialysis buffer (25 mM HEPES, pH 7.4, 150 mM NaCl, 1 mM CaCl_2_) for two days at 4 °C to slowly remove the detergent. Non-reconstituted protein was separated from proteoliposomes by density gradient ultracentrifugation in a 5 mL iodixanol step gradient (OptiprepSTEMCELL Technologies Inc., Vancouver, BC, Canada) (30%, 10%, 5%, 2.5% (*w*/*v*) in dialysis buffer) at 208,000× *g* for 2 h at 4 °C (MLS-50 swinging-bucket rotor, Beckmann Coulter, Brea, CA, USA). The liposome fraction was collected and dialyzed for three days against dialysis buffer. Empty liposomes without any integrin protein were prepared following the same protocol as for the proteoliposomes (adding protein storage buffer instead of purified protein). The reconstitution efficiency was varying between the DOPC:SM:cholesterol ratios and was approximately 27 ± 2% for Ld/Lo, 9 ± 1% for Ld/Lo^low-chol^ and 0.8 ± 0.5% for Lo proteoliposomes.

**Dynamic Light Scattering (DLS)**

DLS measurements were acquired on a Zetasizer Nano ZS and Ultra (Malvern Instruments, Herrenberg, Germany) and data were analysed using the Zetasizer Ultra software. Samples were diluted 1:10 in the respective buffer and 700 µL were transferred to a disposable 10 mm path length macro cuvette (Brand, Wertheim, Germany). After an equilibration time of 2 min at 37 °C measurements were performed at a detector angle of 173°. Each measurement included at least 12 runs for approximately 2 min in total and was repeated three times. Size distributions were calculated using a refractive index of 1.45 and absorption of 0.001 with standard solvent parameters for water. 

**Sodium Dodecyl Sulfate Polyacrylamide Gel Electrophoresis (SDS-PAGE)**

Reducing SDS-PAGE was carried out to detect the integrin subunits of purified and reconstituted proteins. Samples were denatured for 5 min in reducing SDS sample buffer at 95 °C. Then, samples were loaded to 12% Tris-Glycine SDS gels and visualized with Coomassie blue G-250 (Thermo Fisher, Darmstadt, Germany). Commercially available Integrin αIIbβ3 (Enzyme Research Laboratories, South Bend, IN, USA) was used as a control. The molecular weight markers used were Novex™ Sharp Unstained Protein Standard (Invitrogen, Carlsbad, CA, USA) and PageRuler^TM^ Prestained Protein Ladder (Thermo Fischer Scientific, Waltham, MA, USA) for liposome SDS-PAGE and integrin purification SDS-PAGE gels, respectively.

**Lipid Extraction and Thin-Layer Chromatography (TLC)**

TLC was carried out to control the stability and ratio of lipids after reconstitution. Lipids were extracted in a two-step extraction protocol [36]. Liposome or proteoliposome samples were extracted by addition of chloroform:methanol, 10:1 (vol/vol) followed by 20 min incubation on ice and centrifugation for 5 min at 1000× *g* at 4 °C. The organic phase was collected and the polar phase was re-extracted with chloroform:methanol, 2:1 (vol/vol), as described above. Both organic phases were pooled, dried under a stream of nitrogen and the lipids were dissolved again in chloroform: methanol, 2:1 (vol/vol). The sample was spotted on a TLC silica gel 60G plate (Merck KgaA, Darmstadt, Germany) and TLC was performed with chloroform:methanol:H_2_O 65:25:4 (vol/vol/vol) as mobile phase. Lipids were stained with 0.03% Coomassie Blue R-250 in 20% methanol and imaged in an iBright FL1500 Imaging System (Thermo Fisher Scientific, Waltham, MA, USA).

**Transmission Electron Microscopy (TEM)—Negative Stain**

The flotation method was used for the negative staining procedure. Particles were allowed to adsorb onto a glow-discharged carbon-coated Pioloform film on a 400-mesh grid for 5 min. The grid was then transferred sequentially onto two droplets of deionized water, onto a droplet of 2% aqueous phosphotungstic acid (pH 7) for 30 s and finally on a second droplet of 2% aqueous phosphotungstic acid (pH 7) for 4 min. After blotting with filter paper and air-drying, the samples were examined with a transmission electron microscope LEO 906 (Carl Zeiss Microscopy GmbH, Oberkochen, Germany) at an acceleration voltage of 80 kV. For image acquisition, a wide-angle dual speed CCD camera Sharpeye (Tröndle, Moorenweis, Germany) was used, operated by the ImageSP software. Micrographs were processed using Adobe Photoshop CS6. At least three images were taken from two different batches, respectively.

**Activation state assay by QCM-D**

QCM-D measurements were performed as described elsewhere [10] with a Q-sense Analyzer under continuous flow of 25 µL/min operated by a peristaltic pump at 37 °C. For cleaning, SiO_2_-coated quartz crystal sensors were incubated with 2% SDS solution for at least 30 min at RT followed by rinsing with ultrapure water. After drying of crystals under a stream of nitrogen, the surface was exposed for 20 min to UV-ozone. Resonance frequency and dissipation were measured at different harmonics (15, 25, 35, 45, 55, 65 MHz) simultaneously. Changes in frequency (Δ*f*) dissipation (Δ*D*) of the seventh overtone (35 MHz) are displayed in the representative QCM graphs. After equilibration of the system with the respective buffer, liposomes or proteoliposomes were introduced to the system. Vesicle fusion on the SiO_2_ surface was induced using 13 µM α-helical peptide [37] followed by rinsing for at least 20 min with buffer. The integrin activation state was determined after treatment of the bilayer with respective buffer containing 1 mM MnCl_2_ for approximately 30 min. The murine monoclonal IgM antibody PAC-1 (Biolegend, San Diego, CA, USA), which recognizes an activation-induced conformational epitope [38], was added at a concentration of 5 µg/mL for interaction analysis by frequency changes followed by rinsing with the respective buffer (i.e., biobead buffer or dialysis buffer). Frequency and dissipation changes of the 7th overtone between injection time (Δ*f*_7_inject) of PAC-1 and 20 min dissociation time (Δ*f*_7_diss) were calculated and the differences are described as ∆*f* and ∆*D* in the respective graphs. The values were normalized with respect to the protein content of the final proteoliposome batch determined by SDS-PAGE. 

## 3. Results

In order to study integrin activation in specific lipid environments containing cholesterol and sphingomyelin, we applied two distinct protocols to reconstitute integrin αIIbβ3 in model membranes. Those protocols differ fundamentally from each other, in particular concerning the solubilising detergent (i.e., Triton X-100 or CHAPS) and the detergent removal (i.e., SM2 biobeads adsorption or dialysis). In the following, those will be termed “biobead protocol” and “dialysis protocol”. The proteoliposomes generated with those protocols are compared in the following section and have been finally used for integrin activation studies by QCM-D.

### 3.1. Different Reconstitution Procedures of Integrin into Cell Membrane Mimetic Liposome System

The two detergents Triton X-100 and CHAPS feature different chemical properties concerning critical micellar concentration and micelle size and, thus, distinct detergent removal strategies were applied. The detergent Triton X-100, which has been traditionally used for integrin αIIbβ3 purification, was removed by adsorption to SM2 biobeads (as described elsewhere for integrin reconstitution in DMPG and DMPC proteoliposomes [8,10,12]). Protein reconstitution in lipid vesicles was confirmed by DLS, TEM, and SDS-PAGE.

**Figure 1:**
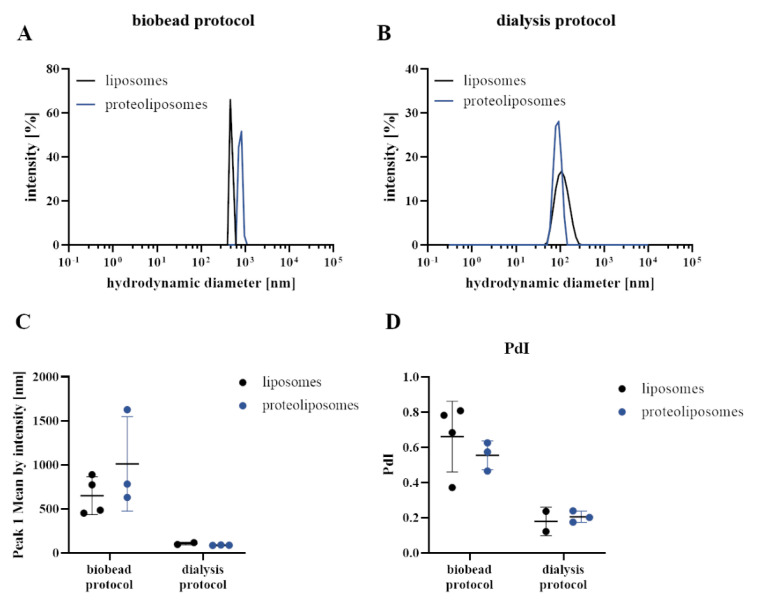
Size distribution of Ld/Lo αIIbβ3 proteoliposomes and empty liposomes reconstituted with different protocols. (**A**) Representative DLS data showing the size distribution of liposomes (black) and proteoliposomes (blue) consisting of DOPC:SM:cholesterol (45:30:25) prepared with the biobead or (**B**) with the dialysis protocol measured at 37 °C. (**C**) Peak 1 mean by intensity and (**D**) polydispersity indices (PdI) from (proteo-)liposome DLS data of at least two independent measurements are shown in dot plots with mean ± SD. Corresponding autocorrelation functions are presented in Figure S2.

**Figure 2:**
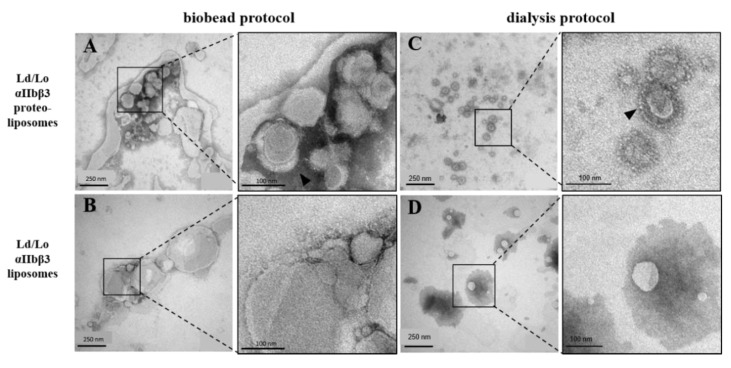
TEM negative stain images of Ld/Lo αIIbβ3 proteoliposomes and empty liposomes containing DOPC:SM:cholesterol (45:35:25 mol%) prepared with different reconstitution protocols. Proteoliposomes (top) and analogously prepared empty liposomes (bottom) were prepared using the (**A**,**B**) biobead protocol or (**C**,**D**) dialysis protocol. Densities corresponding to the characteristic αIIbβ3 extracellular domains are indicated by arrow heads.

Upon detergent removal by SM2 biobeads, large proteoliposomes with a diameter of >500 µm were observed by DLS, in line with the fact that vesicles were not sized by any physical means (Figure 1). The autocorrelation function indicates aggregated particles and a high degree of heterogeneity in size (Figure S2A) with high polydispersity indices (PdI) above 0.45 (Figure 1D), precluding a reliable size analysis of biobead protocol-derived liposomes by DLS. Negative stain TEM images showed some spherical densities but the majority appeared as large (proteo-)liposomes of varying sizes that collapsed on the EM grid or appeared prone to clumping (Figure 2A,B) reflecting our DLS results. Most notably, we were not able to form homogenous lipid bilayers on a crystal for QCM-D studies. The failure to form bilayers on the QCM crystal from proteoliposomes produced by the biobead protocol prompted us to revise our reconstitution strategy. We applied a fundamentally different protocol, which has been successfully used before to reconstitute another single-spanning transmembrane protein, the EGF receptor, in proteoliposomes [35]. In contrast to the biobead protocol (in which lipid films were directly solubilised with detergent-containing buffer) liposomes were first generated from lipid films, transformed into predominantly unilamellar vesicles by freeze-thaw cycles, and sized using a 100 nm filter before solubilisation for reconstitution. Full-length αIIbβ3 was purified from thrombocytes using an adapted protocol from Gingras et al. [9], but included an on-column detergent exchange step to CHAPS (Figure S1). We confirmed the conformational integrity and thermostability of CHAPS-solubilized αIIbβ3 by nanoDSF (Figure S1G), which demonstrates a sharp unfolding transition at 52.66 ± 0.05 °C with the onset of unfolding at 48.37 ± 0.04 °C, which is well above any experimental condition used here. MnCl_2_ addition shifted the unfolding temperature to 54.80 ± 0.01 °C.

For proteoliposome reconstitution, we mixed solubilised αIIbβ3 to CHAPS-destabilized liposomes, and detergent was slowly removed by dialysis. This approach yielded significantly more homogeneous preparations than the biobead protocol. Liposomes and proteoliposomes were more uniform in size and amenable to DLS analysis that demonstrates an average diameter of ~100 nm (Figure 1B) with a lower PdI around 0.24.

The generation of smaller and more homogeneous vesicles was furthermore confirmed by TEM (Figure 2C,D). We visualized the proteoliposomes containing a characteristic integrin corona (Figure 2C, close-up). Approximately 20 nm-long “spike”-like structures with terminal globular heads extended from the rim of the spherical liposomes, which are characteristic for integrins [8,39,40]. As expected, bare liposomes were devoid of such structures (Figure 2D). 

**Figure 3:**
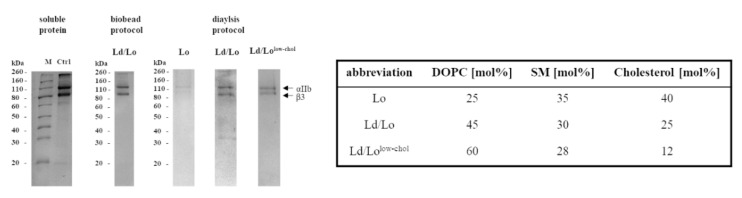
The reconstitution of full-length integrin in several lipid environments as validated by reducing SDS-PAGE. The lipid ratios are specified on the right. Proteoliposomes prepared with the dialysis protocol or the biobead protocol were analysed by SDS-PAGE showing the presence of both integrin subunits with the expected apparent molecular weight of 90 and 110 kDa. Commercial Triton X-100-solubilzed integrin αIIbβ3 (150 µg/mL) was used as control (Ctrl).

The composition of proteoliposomes with respect to lipid and protein content was assessed by thin-layer chromatography (Figure S3) and reducing SDS-PAGE, respectively (Figure 3). The final integrin content appeared lower in dialysis-prepared proteoliposomes, suggesting a lower reconstitution efficiency compared to the biobead protocol (Figure 3). However, DLS and TEM images demonstrated an increased homogeneity without signs of proteoliposome clumping or aggregation. Most importantly, we could successfully form integrin containing bilayers on QCM crystals, which enabled us to study integrin activation. After injection of proteoliposomes, the vesicle rupture events and formation of a homogenous bilayer were observed, indicated by typical changes in frequency and dissipation as described elsewhere [10,23].

**Figure 4:**
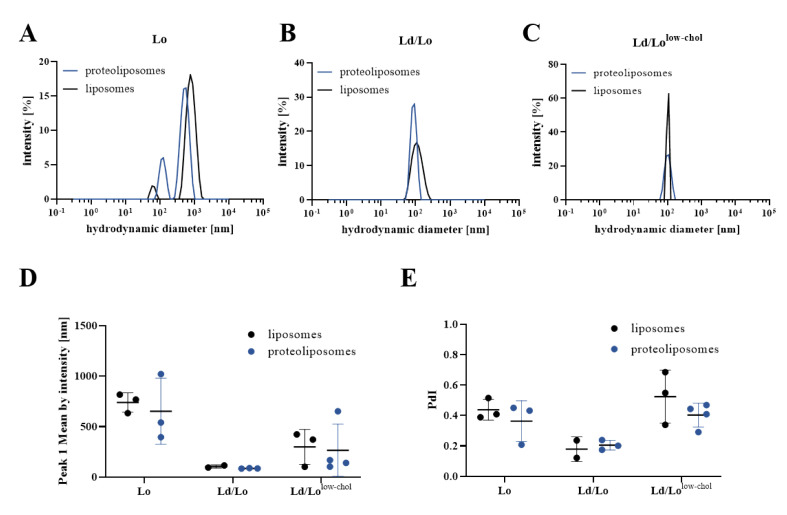
Intensity distribution of (proteo-)liposomes measured by DLS. Representative DLS data obtained at 37 °C show the scattering intensity of liposomes and proteoliposomes prepared with the dialysis protocol containing different DOPC:SM:cholesterol ratios (**A**) Lo, (**B**) Lo/Ld, and (**C**) Lo/Ld^low-chol^. (**D**) Peak 1 mean by intensity and (**E**) polydispersity indices (PdI) from (proteo)liposome DLS data of at least two independent measurements are shown in dot plots with Mean ± SD. Corresponding correlation functions are presented in Figure S4.

Therefore, further experiments to study functionality of integrin were carried out applying the dialysis-based protocol. Various ratios of DOPC:SM:cholesterol were applied to vary the degree of membrane order by different cholesterol amounts, which tuned the system towards more rigid and ordered (Lo) or more fluid membranes (Lo/Ld^low-chol^), specified in Figure 3. Changing the membrane fluidity decreased the efficiency of integrin reconstitution, in particular, the reconstitution of αIIbβ3 in cholesterol-rich (Lo) vesicles was inefficient (Figure 3). Even though the final protein content was low, cholesterol-low proteoliposomes appeared homogeneous in size with approximately 100 nm hydrodynamic diameters (Figure 4) and formed supported-lipid bilayers after deposition on a QCM crystal.

The size distribution of proteoliposomes reconstituted with different DOPC:SM:cholesterol ratios as measured by DLS are displayed in Figure 4. Proteoliposome and empty liposome preparations were approximately 150 nm in size without highly abundant large particles in the micrometer range as seen for proteoliposomes prepared with the biobead protocol. Lo proteoliposome preparations contained some additional particles with a diameter of about 600 nm that scatter intensively (Figure 4A, upper panel), which is reflected by high PdI values of ~0.4. Those larger particles are overrepresented by the intensity distribution, as the scattering intensity is proportional to diameter to the power of six [41]. The autocorrelation functions of liposomes containing low amounts of cholesterol indicate larger aggregates precluding a solid size analysis of Lo/Ld^low-chol^ proteoliposomes. Nevertheless, all those preparations displayed a higher homogeneity when compared to biobead-protocol-derived liposomes. Lo/Ld proteoliposomes display the lowest PdI values below 0.2 and their size could be reliably determined (Figure 4E). 

### 3.2. Effect of Cholesterol Content or Membrane Order on the αIIbβ3 Activation State

**Figure 5:**
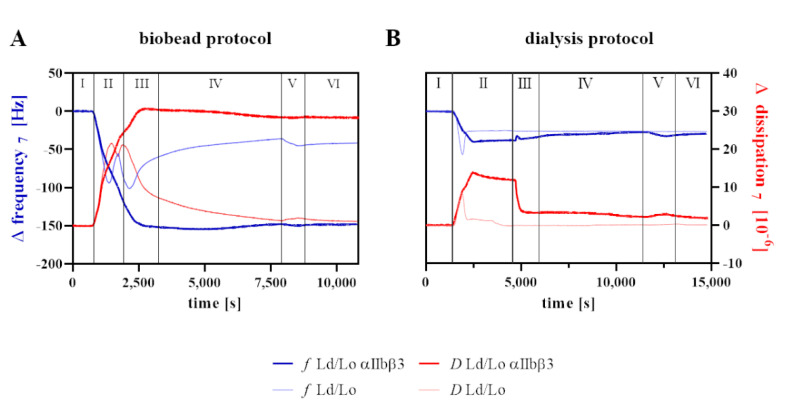
Representative QCM graphs visualizing the experimental setup using Ld/Lo proteoliposomes prepared by the (**A**) biobead or (**B**) dialysis protocol. Changes in frequency (blue) and dissipation (red) of the seventh overtone were recorded at 37 °C. After baseline stabilization (I) liposomes (Ld/Lo-thin line) or proteoliposomes (Ld/Lo αIIbβ3-thick line) were injected (II) and bilayer formation was induced using AH-peptide (III). The bilayer was treated with the respective buffer (i.e., biobead or dialysis buffer ±Mn^2+^) (IV). PAC-1 antibody (5 µg/mL) was injected (V), followed by rinsing with the respective buffer (VI).

**Figure 6:**
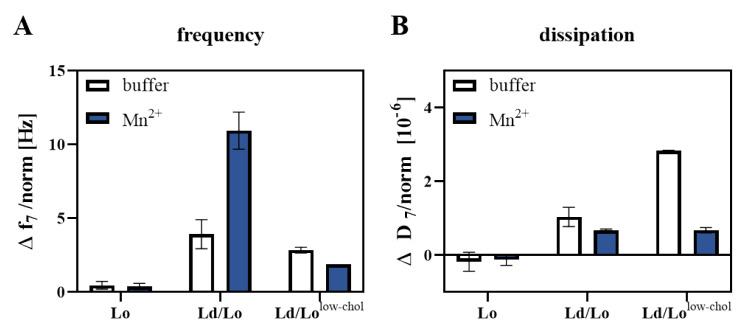
Activation of integrin αIIbβ3 reconstituted in lipid environments of different lipid compositions. The integrin conformation was observed by monitoring the binding of the conformation-specific antibody PAC-1 by QCM-D. DOPC:SM:cholesterol proteoliposomes comprising different ratios of lipids as indicated, were injected on a SiO_2_ surface. Vesicle fusion was initiated by 13 µM AH-peptide. Formed bilayers were equilibrated with 25 mM HEPES, pH 7.4, 150 mM NaCl, 1 m CaCl_2_, with or without 1 mM MnCl_2_ for at least 30 min at 37 °C. Afterwards, PAC-1 antibody (5 µg/mL) was injected for 10 min, followed by rinsing with the respective buffer. Changes in oscillation frequency Δ*f* (**A**) and energy dissipation Δ*D* (**B**) upon PAC-1 injection in QCM-D experiments are shown. Results correspond to two independent measurements ± SEM. Changes in *f* and *D* are normalized to the respective integrin concentration determined by SDS-PAGE. Results for bare liposomes are represented in Figure S5.

The effect of membrane order or microdomain formation on integrin function and specific activation dynamics was addressed using QCM-D. The method provides information about the adsorbed mass (Δ*f*) and the viscoelastic properties (Δ*D*) of the bound material. A representative experimental setup is depicted in Figure 5. After the baseline was reached with buffer (phase I), vesicles were injected (phase II), and fusion was induced by the addition of an α-helical peptide to generate a homogenous bilayer by pore formation and vesicle rupture (phase III). The formed SLB equilibrates at Δ*f* at-30 Hz and Δ*D* near 0 in the absence of transmembrane proteins [37] and at 3 × 10^−6^ for integrin-containing bilayers. The SLB was then rinsed with buffer with or without 1 mM Mn^2+^ (phase IV), which induces the extended integrin conformation corresponding to the activated high-affinity ligand binding state [42]. To monitor this activating conformational transition, the PAC-1 antibody, which exclusively binds αIIbβ3 when extended, was injected (phase V), followed by rinsing with the respective buffer (phase VI). The resulting changes in adsorbed mass (Δ*f*), and viscoelastic properties (∆*D*) upon PAC-1 injection are presented in Figure 6. 

Integrin reconstituted in Ld/Lo or Ld/Lo^low-chol^ bilayers displayed changes in *f* of around 4 Hz upon antibody injection in the absence of Mn^2+^ (Figure 6A) indicating some degree of basal activation. In the presence of divalent Mn^2+^, integrin αIIbβ3 reconstituted in Ld/Lo lipid bilayers was efficiently activated and transitioned to its extended conformation as observed by the high changes in *f* upon PAC-1 injection (up to 12 Hz) (Figure 6). In contrast, Δ*f* remained almost at the same level as the buffer control for αIIbβ3 reconstituted in Lo/Ld^low-chol^ liposomes. Corresponding changes in *D* are lower than 3 × 10^−6^ indicating a low- change in viscoelastic properties. We could not detect any changes in frequency or dissipation after PAC-1 antibody injection to integrin reconstituted in Lo lipid bilayers regardless of Mn^2+^ addition (Δf < 1 Hz, ∆D ~0). However, we cannot exclude that the lack of observable PAC-1 binding might simply be a direct consequence of the reduced integrin reconstitution efficiency in Lo proteoliposomes and therefore below the detection limit. As expected, integrin-free bilayers do not show significant PAC-1 binding under any condition as no significant frequency or dissipation changes were detected (Figure S5). 

## 4. Discussion

Reducing the complexity and dynamics of living cells by bottom-up in vitro reconstitution enables the understanding of biological processes and specific mechanisms at the molecular level. We aimed at establishing a reconstitution approach to study αIIbβ3 conformational dynamics in biomimetic lipid environments. We report a protocol to produce proteoliposomes containing αIIbβ3 in a ternary lipid environment mimicking cellular membranes that form homogeneous supported-lipid bilayers on quartz crystals amenable to QCM-D activation studies, which demonstrates the functionality of reconstituted integrin. The generation of such membrane models, composed of unsaturated phosphatidylcholine and the major plasma membrane lipids cholesterol and sphingomyelin, involves several practical challenges [25]. The selective interactions among sphingolipids and cholesterol within membranes lead to their segregation from unsaturated lipids in model membranes generating immiscible liquid-ordered and liquid-disordered domains [29,43]. The formation of such domains has consequences for their solubility by detergent and might generate highly heterogeneous vesicles with respect to their lipid content and properties [19,33]. Moreover, protein to lipid ratio might affect phase behaviour [44], which needs to be considered in future studies. Another, more general issue, concerns stability as lipids are prone to degradation by hydrolysis and oxidation in an aqueous environment [45,46]. 

These properties pose major challenges on reconstitution approaches as they affect homogeneity and solubilisation properties, which are directly related to reconstitution success and the quality of membrane models [18,19,28]. We were able to embed the αIIbβ3 transmembrane receptor into membrane environments with distinct physicochemical properties. We used lipid compositions, for which phase diagrams have been generated in the past [29,30,31], however, we cannot rule out that the transmembrane proteins affected the phase behaviour. Notably, the proteoliposome reconstitution efficiency varied considerably, and was heavily reduced with cholesterol-rich lipid compositions, which might reflect the partitioning behaviour of αIIbβ3 integrins in platelet membranes [47]. However, it should be noted that the integrin reconstitution efficiency might be further optimized by adapting the detergent amount or the initial lipid:protein ratio [32]. In general, the reconstitution efficiency of a transmembrane protein depends on multiple factors, such as temperature, lipid composition, detergent and the method of its removal and requires empiric optimization [18,48]. Our adapted reconstitution protocol based on liposome sizing by freeze-thaw cycles and extrusion, CHAPS solubilisation, and slow detergent removal by dialysis enabled us to generate homogeneous αIIbβ3 vesicles that formed supported-lipid bilayers for QCM measurements even with such demanding ternary lipid environments. This procedure may be also applicable to other single-spanning or multispanning transmembrane proteins. The usage of a tethered supported lipid bilayer should be considered if support effects are problematic, in particular, with proteins containing large domains that would face the quartz surface [49]. Additionally, avidity effects induced by receptor clustering cannot be excluded and need further investigation by, e.g., imaging of the QCM crystals.

The potential role of the lipid environment on integrin function has been a topic of several studies using, for instance, cell adhesion assays, microscopy techniques and isolation of detergent-resistant membrane fractions [50,51,52,53,54,55]. Our established QCM-D measurement set-up was used to probe the effect of different lipidic environments on αIIbβ3 activation. We monitored the binding of the conformation-selective ligand-mimetic monoclonal antibody PAC-1 as an approximation for integrin activation [38,56]. αIIbβ3 was specifically activated in Ld/Lo environments by Mn^2+^ binding at a remarkably higher level as compared with previous work using αIIbβ3 reconstituted in short-chain DMPG:DMPC bilayers [10] indicating the importance of selecting appropriate membrane models. In contrast, specific activity diminishes in membranes containing lower (Ld/Lo^low-chol^) or high cholesterol amounts (Lo), although these effects might have to be attributed to an overall lower protein content within these membranes. Further optimization is needed to ensure constant protein amounts in those distinct membrane systems. Curiously, while αIIbβ3 embedded in Ld/Lo and Ld/Lo^low-chol^ membranes display some basal activation, only αIIbβ3 reconstituted in Ld/Lo membranes was specifically activated by Mn^2+^.

Our data hint at a relevance of the lipid environment on αIIbβ3 activation. Membrane thickness, hydrophobic mismatch, lateral pressure, and fluidity directly affect the rotational and lateral freedom and interactions between the transmembrane domains, which are crucial for integrin extension and activation [2,4,6]. How exactly these effects translate to integrin activation and signalling remains unclear.

Bodin et al. [47], demonstrated that only a minor fraction of integrin partitions into highly ordered microdomains in platelets irrespective of ligand stimulation, which may be also reflected by our low reconstitution efficiency in a cholesterol-enriched “raft-like” environment (Lo). Nevertheless, the absence of rafts seems to impair integrin signalling and membrane cytoskeleton interactions mediated by integrin lateral tension in platelets [47,57]. How exactly the lipid environment might tune integrin signalling remains a matter of speculation. Here, we demonstrate a well-controlled in vitro reconstitution approach that will be applicable to further studies to understand general integrin (e.g., αVβ3 or LFA-1) dynamics in health and disease. Disease-relevant integrin mutations and binding to distinct ligands could be assessed in a lipid-specific context in basic research as well as for drug screening. This approach is not restricted to integrin-ligand interactions, but may also probe protein-protein interactions in a specific membrane context, e.g., for gaining insights into the development of immunogenicity of αIIbβ3 in immune thrombocytopenia patients. 

## Data Availability

Not applicable.

## Supplementary Materials

The following are available online at https://www.mdpi.com/article/10.3390/membranes11070499/s1, Figure S1: Purification of integrin αIIbβ3, Figure S2: Autocorrelation functions of DLS measurements, Figure S3: Thin-layer chromatography of lipids extracted from liposome samples, Figure S4: Autocorrelation functions of proteoliposomes and empty liposomes with different membrane compositions. Figure S5: QCM control measurement of bare lipid bilayers comprising of different lipid ratios during PAC-1 injection.
