# Supplementary material for: Reconstitution of Functional Integrin αIIbβ3 and Its Activation in Plasma Membrane-Mimetic Lipid Environments
Source: Membranes (Basel). 2021 Jun 30;11(7):499. doi: 10.3390/membranes11070499 (PMC8304682; doi:10.3390/membranes11070499)
Supplement: Supplementary file 1 [file membranes-11-00499-s001.zip › membranes-1272705-supplementary.pdf]

Supplementary Materials

Reconstitution of functional integrin αIIbβ3 and its activation in plasma membrane-mimetic lipid environments

Una Janke[1,2,3], Alexandra Mitlehner[1,2,] Aileen Weide[1,2], Theresia Gutmann[4], Mihaela Delcea[1,2,3*]

[1]Institute of Biochemistry, University of Greifswald, Felix-Hausdorff- Straße 4, 17489 Greifswald, Germany

[2]ZIK HIKE- Zentrum für Innovationskompetenz "Humorale Immunreaktionen bei kardiovaskulären Erkrankungen", University of Greifswald, Fleischmannstraße 42, 17489 Greifswald, Germany

[3]DZHK (German Centre for Cardiovascular Research), Partner Site Greifswald, Greifswald, Germany

[4]Paul Langerhans Institute Dresden of the Helmholtz Zentrum München at the University Hospital and Faculty of Medicine Carl Gustav Carus of TU Dresden, 01307 Dresden, Germany

*Corresponding author: Prof. Dr. Mihaela Delcea, delceam@uni-greifswald.de

Figure S1. Purification of integrin αIIbβ3.

Figure S2. Autocorrelation functions of DLS measurements.

Figure S3. Thin-layer chromatography (TLC) of lipids extracted from liposome samples

Figure S4. Autocorrelation functions of proteoliposomes and empty liposomes with different membrane compositions.

Figure S5. QCM control measurement of bare lipid bilayers comprising of different lipid ratios during PAC-1 injection.

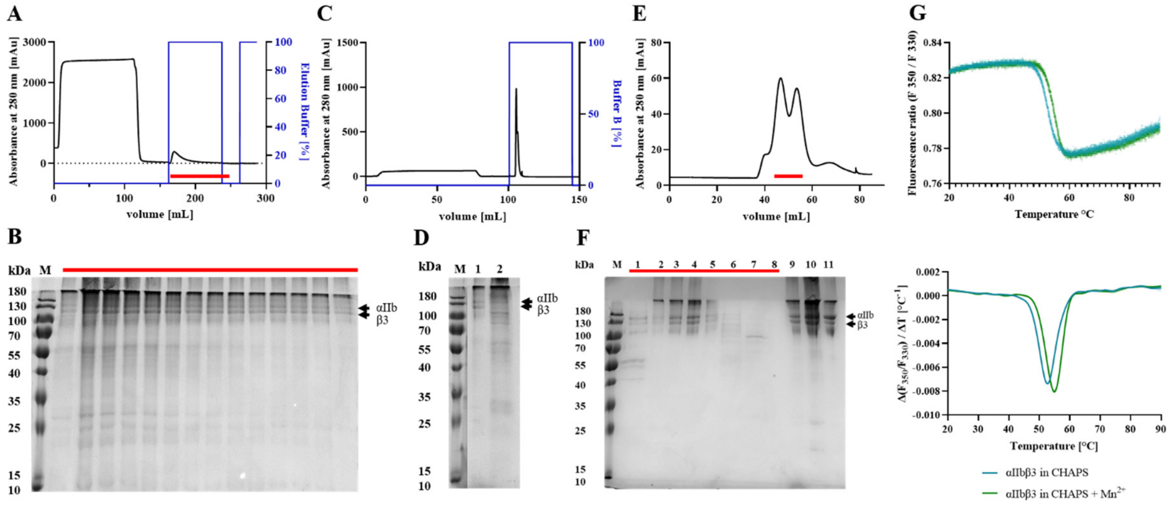

Figure S1. Purification of integrin αIIbβ3. (A) HiTrap Con A 4B 5mL elution chromatogram (black) eluted with 20 mM HEPES, 150 mM NaCl, 1 mM $CaCl_2$, 1 mM $MgCl_2$, 1 % CHAPS, 5 µmol Leupeptin, 200 mM methyl α-D mannopyranoside at a flow rate of 1 mL/min . Collected elution fractions are indicated by the red line. **(B)** Reducing SDS-PAGE of ConA elution factions indicated by the red line and protein molecular weight standard (M) shown on the left. The integrin subunits are indicated by arrows. **(C)** HiTrap Heparin HP 5 mL column chromatogram (black) eluted with 20 mM Tris and 1M NaCl at a flow rate of 1 mL/min. **(D)** Reducing SDS-PAGE of heparin column elution fractions. Protein molecular weight marker (M), flow-through (1) and elution fraction (2) are presented and the integrin subunits are indicated by arrows. **(E)** Size-exclusion chromatogram of the concentrated heparin flow through operated with a flow rate of 0.5 mL/min on a HiPrep 16/60 Sephacryl S-300. The collected elution fractions are indicated by the red line. **(F)** Reducing SDS-PAGE of size-exclusion chromatography elution factions and protein molecular weight standard (M) shown on the left; elution fractions (1-8) indicated by a red line, concentrated heparin FT (9-10) and integrin control (11) are displayed. The integrin subunits are indicated by arrows. (**G**) Thermal stability was assessed by nanoDSF monitoring the intrinsic protein fluorescence at 350 and 330 nm in a temperature ramp (1 °C/min) in the absence or presence of $MnCl_2$. The ratio of fluorescence at 350 and 330 nm is plotted over the temperature (top) and the first derivative is shown below, which was used to determine the melting temperature. Data are shown for 3 technical replicates plotting all values (top) or the mean and transparent error bars representing the standard deviation (bottom).

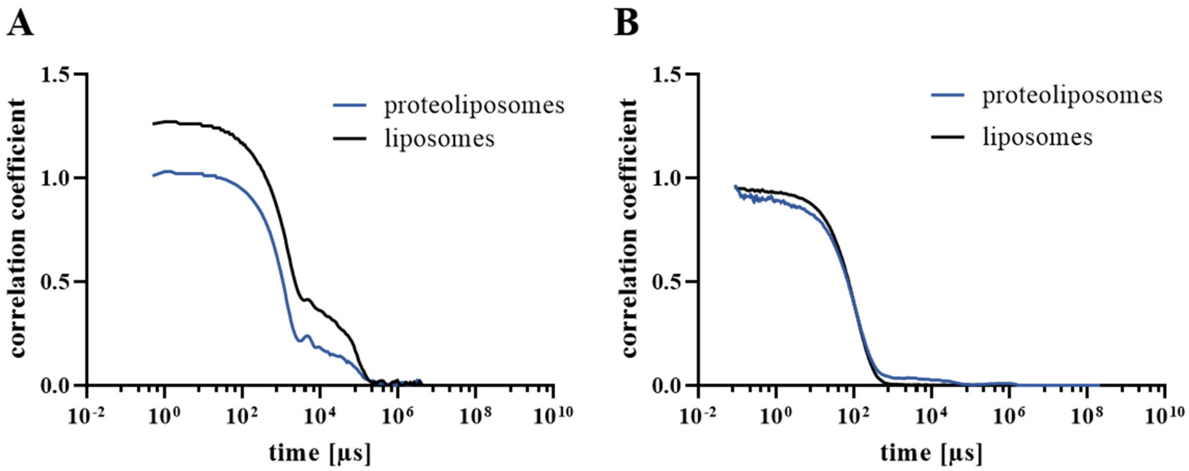

Figure S2. **Autocorrelation functions of DLS measurements. (A)** Correlograms of Lo liposomes (black) and proteoliposomes (blue) prepared following biobead protocol. **(B)** Correlograms of Lo liposomes (black) and proteoliposomes (blue) prepared using dialysis protocol.

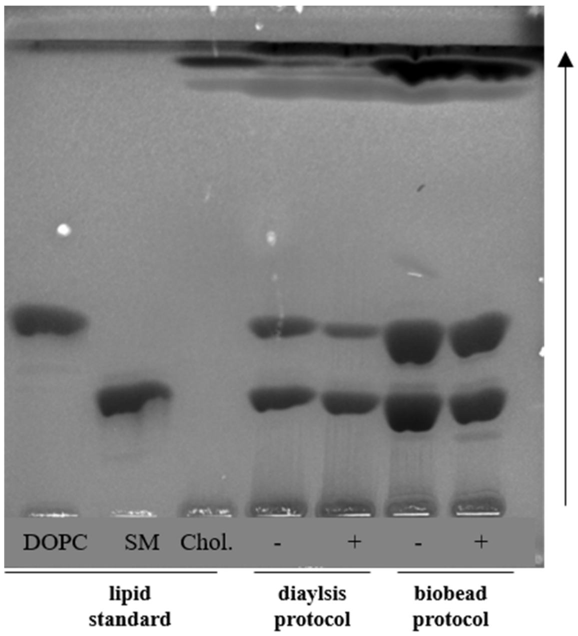

Figure S3. Thin-layer chromatography (TLC) of lipids extracted from liposome samples. 0.5 μmole of DOPC, SM and cholesterol standards are spotted on the plate and are shown on the left. 0.5 μmol lipids from biobead protocol- or dialysis protocol derived (-) liposomes and (+) proteoliposomes were spotted on the TLC plate and plate was stained with 0.03 % Coomassie Brilliant Blue R-250 in 20% methanol.

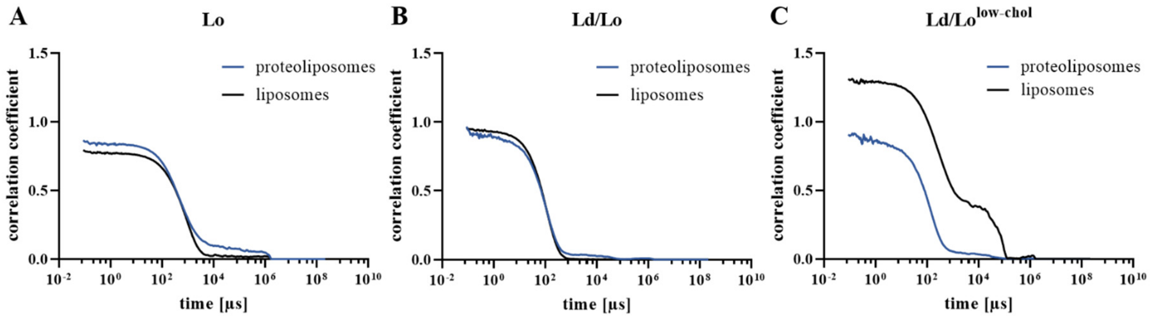

Figure S4. **Autocorrelation functions of proteoliposomes and empty liposomes with different membrane compositions.** Correlogram of **(A)** Lo, **(B)** Ld/Lo, and **(C)** Ld/Lo$^{low-chol}$ liposomes (black) and proteoliposomes (blue) prepared following dialysis protocol.

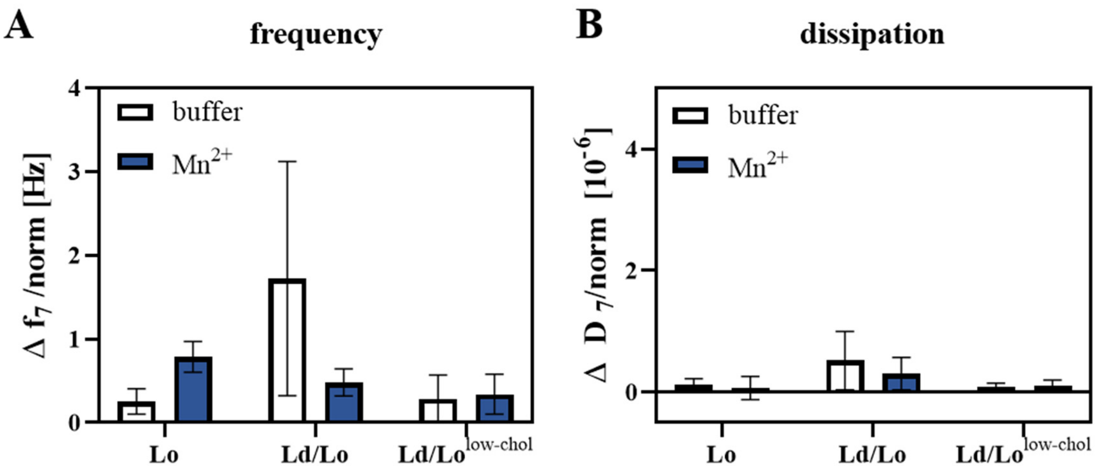

Figure S5. QCM control measurement of bare lipid bilayers comprising of different lipid ratios during PAC-1 injection. Changes in frequency Δf **(A)** and dissipation ΔD **(B)** upon PAC-1 injection event in QCM-D experiments with bare liposomes after treatment with buffer (white) or 1 mM Mn^{2+} (blue) for at least 30 min at 37°C. DOPC:SM:cholesterol liposomes comprising different lipid ratios as indicated in the graphs, are injected on a SiO_2 surface. The vesicle fusion was initiated by 13 µM AH-peptide. Formed bilayer was treated with dialysis buffer with or without 1 mM $MnCl_2$. Afterwards, PAC-1 antibody (5 µg/mL) was injected for 10 min, which was followed by rinsing with the respective buffer. Results correspond to at least 2 independent measurements ± SEM.
